# Assessment of the quality of a software application for the prevention of skin lesions in newborns*

**DOI:** 10.1590/1518-8345.3711.3352

**Published:** 2020-09-07

**Authors:** Simone Vidal Santos, Flávia Regina Souza Ramos, Roberta Costa, Luís Manuel da Cunha Batalha

**Affiliations:** 1 Universidade Federal de Santa Catarina, Hospital Universitário, Florianópolis, SC, Brazil.; 2 Universidade Federal de Santa Catarina, Departamento de Enfermagem, Florianópolis, SC, Brazil.; 3 Escola Superior de Enfermagem de Coimbra, Unidade de Investigação em Ciências da Saúde: Enfermagem, Coimbra, Portugal.

**Keywords:** Neonatal Nursing, Infant, Newborn, Skin, Nursing Care, Mobile Applications, Technology Assessment, Biomedical, Enfermagem Neonatal, Recém-Nascido, Pele, Cuidados de Enfermagem, Aplicativos Móveis, Avaliação da Tecnologia Biomédica, Enfermería Neonatal, Recién Nacido, Piel, Atención de Enfermería, Aplicaciones Móviles, Evaluación de la Tecnología Biomédica

## Abstract

**Objective:**

to assess the technical quality of a mobile application to support the nurse’s decision to prevent skin lesions in hospitalized newborns, according to the Product Quality Model.

**Method:**

a methodological study for technological assessment. The 20 evaluators, divided into two groups, 10 nurses and 10 information technology professionals, used the software*,* conducted tests based on two case studies, and evaluated six features and 23 sub-features of quality. The assessment was conducted by means of an online form. Data was analyzed through a specific formula and the items that obtained a concordance percentage over 70% were considered adequate.

**Results:**

the concordance percentages of the features in the groups of nurses and of information technology specialists were the following: functional adequacy (100%-98.9%), reliability (90%-100%), usability (93.2-85%), performance efficiency (100%-100%), compatibility (97.5-90%), and safety (94%-91%). In the assessment of the sub-features, only accessibility presented a percentage value below the desired one (70%-60%).

**Conclusion:**

the software has excellent technical quality to meet the needs of nurses in planning the care for the prevention of skin lesions of hospitalized newborns, brings important advances to neonatal care, contributes to the work process, expands knowledge, and promotes the professional’s clinical reasoning.

## Abstract (pt)

**Objetivo:**

avaliar a qualidade técnica de um aplicativo móvel para apoiar a decisão do enfermeiro nos cuidados de prevenção de lesões de pele em recém-nascidos hospitalizados, de acordo com o Modelo de Qualidade do Produto.

**Método:**

estudo metodológico de avaliação tecnológica. Os 20 avaliadores, divididos em dois grupos, 10 enfermeiros e 10 profissionais da tecnologia da informação, utilizaram o *software,* realizaram testes a partir de dois estudos de caso e avaliaram seis características e 23 subcaracterísticas de qualidade. A avaliação foi realizada por formulário online. Os dados foram analisados através de fórmula específica e foram considerados como adequados os itens que obtiveram percentual de concordância superior a 70%.

**Resultados:**

os percentuais de concordância das características pelo grupo de enfermeiros e de tecnologia foram: adequação funcional (100%-98,9%), confiabilidade (90%-100%), usabilidade (93,2-85%), eficiência de desempenho (100%-100%), compatibilidade (97,5-90%), e segurança (94%-91%). Na avaliação das subcaracterísticas, apenas a acessibilidade apresentou percentual abaixo do desejado (70%-60%).

**Conclusão:**

o *software* apresenta excelente qualidade técnica para atender às necessidades do enfermeiro no planejamento dos cuidados de prevenção de lesões de pele dos recém-nascidos hospitalizados, traz importante avanço para a assistência neonatal, contribui para o processo de trabalho, amplia conhecimentos e fomenta o raciocínio clínico do profissional.

## Abstract (es)

**Objetivo:**

evaluar la calidad técnica de una aplicación para dispositivos móviles destinada a sustentar las decisiones del enfermero con respecto a los cuidados para prevenir lesiones cutáneas en recién nacidos internados, de acuerdo con el modelo de Calidad del producto.

**Método:**

estudio metodológico de evaluación tecnológica. Los 20 evaluadores, divididos en dos grupos (uno de 10 enfermeras y otro de 10 profesionales en Tecnología de la Información), utilizaron el *software,* realizaron pruebas a partir de dos estudios de casos, y evaluaron seis características y 23 subcaracterísticas de calidad. La evaluación se realizó por medio de un formulario en línea. Los datos se analizaron a través de una fórmula específica y se consideraron como adecuados los ítems que obtuvieron un porcentaje de acuerdo superior al 70%.

**Resultados:**

los porcentajes de acuerdo de las características que se obtuvieron en el grupo de enfermeras y en el de especialistas en Tecnología de la Información fueron los siguientes: adecuación funcional (100%-98,9%), confiabilidad (90%-100%), facilidad de uso (93,2-85%), eficiencia de rendimiento (100%-100%), compatibilidad (97,5-90%) y seguridad (94%-91%). En la evaluación de las subcaracterísticas, solamente la accesibilidad presentó un porcentaje por debajo del deseado (70%-60%).

**Conclusión:**

el *software* presenta excelente calidad técnica para responder a las necesidades de los enfermeros en cuanto a la planificación de los cuidados para prevenir lesiones cutáneas en recién nacidos internados, aporta un importante avance para la asistencia neonatal, contribuye al proceso de trabajo, expande conocimientos, y fomenta el raciocinio clínico de los profesionales.

## Introduction

Technological advances have made the survival of ever smaller and more premature newborns (NBs) possible, evidencing the problems with skin integrity in the Neonatal Unit (NU), largely caused by the anatomical and physiological specificities of the neonate and by the need to use devices, indispensable for the care of these patients. This scenario has been responsible for concerns of the neonatal nurses regarding skin care for NBs^(^ 1 ^)^. 

In an NU, there are few interventions that do not present risks, and nurses need to know how to identify products and procedures that can be useful, as well as the unintended negative consequences that an intervention can cause^(^ 2 ^)^. 

In view of the structural specificities of the newborn’s skin, the potential for lesions is high^(^ 3 ^)^. The lower the gestational age (GA), the higher the risk^(^ 4 ^)^. These lesions can increase the chances of infections, main cause of neonatal morbimortality, in addition to provoking definite scars and functional alterations^(^ 5 ^-^ 6 ^)^. They also contribute to longer hospital stays and to increased costs of hospitalization^(^ 7 ^)^. Therefore, although it is an area of clinical practice that still needs to be improved, the prevention of skin lesions is fundamental in the care of NBs^(^ 8 ^-^ 10 ^)^. 

The integrity of the integument should be used as a metric to leverage the quality of neonatal care^(^ 1 ^)^ and, with regard to the performance of their praxis, nurses must acquire the necessary knowledge in order to improve the care provided to the NB. For this, they can use technologies that offer adequate information and that assist in the decision-making process.

The use of technologies in the health field has grown considerably and collaborates with the monitoring of the health of individuals in an effective and personalized way^(^ 11 ^)^. Clinical decision support systems (CDSSs) allow adopting prevention and early diagnosis strategies, thus improving the quality of patient care^(^ 12 ^)^. 

In order to combine technology and care in the neonatal area and contribute to support the decision in the prevention of skin lesions in newborns, *Neonatal Skin Safe*? was developed, an application for mobile devices developed by nurses. The development of this technology is justified by not knowing about the existence of any software for mobile devices that can be used by the neonatal nurse at the bedside and that has the function of targeting the skin care of the hospitalized NB, considering the anatomical, physiological, and care conditions. In this sense, this study brings important contributions to improve the care and safety of neonatal patients.

The content of *Neonatal Skin Safe*? presents evidence-based recommendations, which have also been validated by the experts on the theme in question^(^ 13 ^)^. However, to ensure that the software presents the necessary requirements to meet the needs of the users, its quality needs to be assessed. The quality of software ensures that the product presents the features, functions, and content that the user wants, offering agility in carrying out the processes for which it was developed^(^ 14 ^)^. 

In this sense, evaluating the quality of *Neonatal Skin Safe*? becomes an important procedure as it aims to ensure that the software performs its functions quickly and safely, as well as that it provides correct information and instructs the nurse as to the proper care of the newborn’s skin, in order to prevent risks that may cause iatrogenesis.

There are several methodologies for assessing the quality of software applications. The Brazilian Association of Technical Standards (*Associação Brasileira de Normas Técnicas*, ABNT), based on the International Organization for Standardization (ISO) and together with the International Electrotechnical Commission (IEC), offers ISO/IEC 25010-2011:*System and Software engineering*- (*SquaRE)*- System and software quality models^(^ 15 ^)^, which deals with the quality of software*,* and ISO/IEC 25040-2011: *System and Software engineering*- (*SquaRE)*- Evaluation process^(^ 16 ^)^, which provides guidance on the evaluation process.

Considering the need to assess its technical quality and ensure that the software meets the needs of users, the following guiding question was elaborated: Does a mobile application developed to guide in relation to care for the prevention of skin lesions in hospitalized NBs present technical quality for its use by neonatal nurses? The aim of this study was to assess the technical quality of the *Neonatal Skin Safe*?, a mobile application to support the nurse’s decision to prevent skin lesions in hospitalized NBs, according to the Product Quality Model^(^ 15 ^)^.

## Method

A methodological and quantitative study for technological assessment. This type of study deals with the development, validation, and evaluation of tools, and is generally focused on developing new instruments^(^ 17 ^)^. The assessed software is an application for mobile devices that was named *Neonatal Skin Safe*?. It was developed from July 2017 to December 2018, from an integrative literature review^(^ 18 ^)^, content validation, and technical development. 

The evaluation of the technical quality of the application was carried out according to the product quality evaluation process of software, ISO/IEC 25040-2011^(^ 16 ^)^, which guides five stages: (1) Establishing the evaluation requirements; (2) Specifying the evaluation, defining the quality metrics, the scoring levels, and the criteria for judgment; (3) Designing and planning the evaluation activities; (4) Conducting out the evaluation, carrying out measurements, applying decision criteria for quality measures and for evaluation; and, (5) Completing the evaluation. 

The ISO/IEC 25010-2011 standard divides the quality model in two stages, which can be applied in any type of software, namely: (A) Product Quality and (b) Quality in Use^(^ 15 ^)^. 

The Product Quality Model identifies eight quality features, namely: functional adequacy, performance efficiency, compatibility, usability, reliability, safety, maintainability, and portability. Each of these features has its own sub-features, for a total of 31^(^ 15 ^)^. 

Quality in Use identifies the following as quality requirements: efficacy, efficiency, satisfaction, risk-free operation, and context coverage. The “satisfaction” feature presents the following sub-features: usefulness, confidence, pleasure, and comfort. “Risk-free operation” has the following sub-features: reduction of the economic risk, reduction of the health and safety risk, and reduction of the environmental risk. Finally, “context coverage” has integrality of context and flexibility as sub-features^(^ 15 ^)^. 

**Figure 1:**
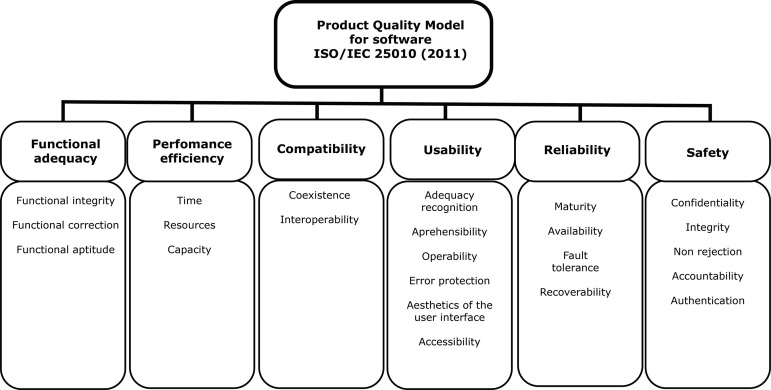
Features and sub-features of the Product Quality Model of the software used to assess *Neonatal Skin Safe*?. Adapted from ABNT ISO/IEC 25010 (2011) - Free translation by the author. Florianópolis, SC, Brazil, 2019

As regards the evaluation requirements of the software, it was decided to establish the Product Quality Model, for considering that it groups properties which are relevant to the object being evaluated. This model analyses eight quality features; however, as it is not possible to provide the source code of the software to the evaluators, it was decided to evaluate only six of the eight quality features, excluding maintainability and portability^(^ 15 ^)^, as shown in Figure 1. 

To proceed with the evaluation process, it was initially defined that the software would be assessed by three groups: (1) assistential nurses; (2) teacher-nurses; and, (3) professionals working in the Information Technology (IT) area with experience in the development of software. However, due to the low adherence of the teacher-nurses, it was decided to work only with two groups: (1) assistential nurses and/or teacher-nurses and/or stomatherapists, with experience in caring for NBs; and (2) IT professionals and/or professors. 

The number of participants followed the guidelines of ABNT/ISO/IEC 25062-2011, which recommend a minimum sample of eight evaluators for each category of users, in order to ensure representativeness in relation to the intended user group^(^ 19 ^)^. 

The search for evaluators, both in the IT area and in the nursing area, was carried out through the Lattes Platform of the National Council for Scientific and Technological Development (CNPq), using advanced research by subject matter in order to facilitate the identification of Brazilian professionals with expertise in the theme under study. To select stomatherapist nurses, the Brazilian Society of Stomatherapy (*Sociedade Brasileira de Estomaterapia*, SOBEST) was also used as a resource to identify professionals with experience in the neonatal area. 

The inclusion criteria used to select the experts were adapted from a scoring system for validation studies^(^ 20 ^)^. Titles, the participant’s experience in the study area, and the scientific production were considered. Each specialist should obtain a minimum score of five points, according to their area/specialty, as shown in Figure 2.

**Figure 2:** Scoring system for the selection of the evaluators. Adapted from Fehring (1987). Florianópolis, SC, Brazil, 2019

Inclusion Criteria	Score
Graduated in Nursing for at least 2 years or Bachelor of Computer Science for at least two years	2
Experience in teaching or neonatal care for at least 2 years or experience in the development of software for at least two years	3
Master's degree in Nursing with a dissertation in the field of neonatal nursing and/or Specialist in Stomatherapy with emphasis on neonatal/pediatric care or Master's degree in Computer Sciences, with a dissertation on software development	2
PhD in Nursing with a thesis on the skin theme or PhD in Computer Sciences with a thesis on software development	3
Research studies published with an emphasis on the skin theme or research studies published on software development	2
Participating in a research laboratory in the field of neonatal nursing and/or stomatherapy or teaching experience in the area of software development	1

The exclusion criterion established was having been away from work activities for a period of more than two years. 

The professionals were invited through an invitation letter sent by email. The participants who answered positively to the invitation were asked to read and sign the Free and Informed Consent Term (FICF), as well as to provide information about the operating system used in their smartphones. Soon after, they received the guidelines for carrying out the evaluation, the link to download the software, two case studies of fictional patients for each evaluation group, and the link to access the evaluation form. 

The evaluation was carried out using two forms from *Google Forms*?, one for the group of nurses and another for the group of IT specialists, containing the features and specific questions for the quality sub-features. They were adapted from a previous study^(^ 21 ^)^; thus, the data collection instruments were already validated, minimizing the chances of bias in this study.

Data collection was conducted from December 2018 to January 2019. The participants were given seven days to complete the evaluation form, and it was necessary to extend the period for another five days, in order to ensure that the evaluators finished the process. During this period, the researcher was available to answer questions and help the participants via email, phone, and *WhatsApp*.

To specify the assessment, the quality metrics, scoring levels, and criteria for judging the software were defined^(^ 16 ^)^. Each evaluator attributed a score level to each of the assessed sub-features. The score levels were defined as follows: (A) Agree; (D) Disagree; (NA) Not Applicable; Comments^(^ 22 ^)^. The specialists’ comments, especially when accompanied by a level D (Disagree) score, were important to unveil the items that need improvement in the software. 

The data on the answers to the key questions from the evaluation forms of each participant were tabulated in *Microsoft Excel* spreadsheets, analyzed by absolute (n) and relative (%) frequency in *Statistical Package for Social Sciences*(SPSS) 20.0, and divided by evaluation group. The mean and standard deviation (SD) of the values of the quality sub-features were calculated based on the exclusion of the “Not applicable” answers for each evaluator. The values expressed in percentages of the quality features evaluated were obtained through the adapted formula^(^ 21 ^-^ 22 ^)^, where: the measured value of the feature (Vc) (=) the sum of the value of the sub-features with an Agree answer (?Vsca) (?) Agree answers (a) (+) Disagree answers (d) (+) Not applicable answers (na) (-) Not applicable answers (na) (x) 100. SPSS was also used to analyze quality features and sub-features.

For analyzing the results, the items that obtained a percentage of agreement greater than 70% were considered adequate, according to the rating scale for sub-features^(^ 21 ^-^ 22 ^)^, where 25% (weak); 50% (regular); 75% (good); and 100% (excellent). 

Data was presented by means of tables and in a descriptive manner. The research study met the ethical precepts of Resolution 466/12 of the National Health Council. Its development was approved by the Research Ethics Committee of the Federal University of Santa Catarina, under opinion No. 2,229,207, and CAAE: 69500917.1.0000.0121. The participants were guaranteed confidentiality, anonymity, freedom of participation, and the possibility to withdraw at any time.

## Results

*Neonatal Skin Safe*? is a tool that contributes to the identification of anatomical, physiological, and treatment-related conditions of the hospitalized NB and which may lead to risk for skin lesions, and to the nurse’s decision making regarding these risks.

The software is available as a free download in the *App Store*? and in *Google Play*?, being compatible with smartphones and tablets operating with iOS and Android? technologies, which can be found using the search tool on these platforms by typing the name *Neonatal Skin Safe*?. To perform the download, the user needs Internet access; however, after being saved in the memory of the user’s smartphone or tablet, the application will also be available for off-line use.

The home screen of the application requires nurses to register with the data of their full name, number of the Regional Nursing Council (*Conselho Regional de Enfermagem*, COREN), and state, as well as to create an access password, so that the data is protected on their devices. The second screen welcomes the users and provides information about the construction, content, and navigation of the application. The following screen offers a brief explanation of the anatomy, physiology, and risks of skin lesions to which hospitalized NBs are exposed.

The fourth screen presents a patient registration form that includes the following fields to be completed: Identification (number assigned by the user or medical record number), NB name (initials), mother’s name (initials), date of birth, time of birth, GA at birth (weeks + additional days), and birth weight (expressed in grams). On this screen, nurses must also answer whether the NB’s mother is infected with the Human Immunodeficiency Virus (HIV) or with Hepatitis B.

From the fifth screen, with the NB’s Skin Condition Scale (*Escala de Condição da Pele do RN*, ECPRN) that assesses dryness, erythema, and rupture/lesion^(^ 23 ^)^, the neonatal assessment begins, followed by the neurological condition (alertness and physical mobility of the newborn), oxygenation (use of devices for respiratory support), hydration (edema and dehydration), nutrition (use of devices for feeding), thermal regulation (thermal regulation capacity of the newborn), ambience (incubator or cradle), route for drug therapy (subcutaneous, intramuscular, intravenous), drug therapy (medications in use: antibiotics, hyperosmolar agents, vasoactive drugs, and sedatives, among others), umbilical stump (presence of the stump and catheterization), monitoring (oximetry, cardiac monitor, blood pressure cuff), urinary elimination (spontaneous diuresis, relief vesical catheter, delayed vesical catheter, cystostomy, urostomy), intestinal elimination (normal, altered, ostomy), and others (phototherapy, chest drain, peritoneal drainage, and surgical wound). 

**Figure 3:**
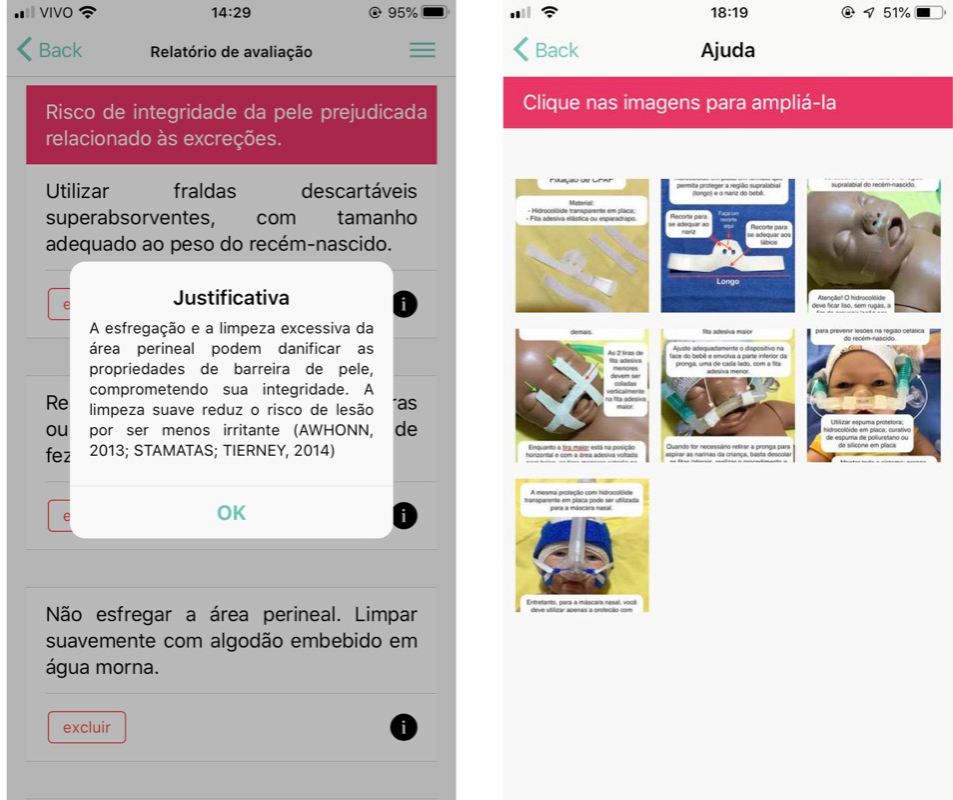
Diagnosis, nursing interventions, justifications, and photographs screens with step-by-step instructions - *Neonatal Skin Safe*?. Florianópolis, SC, Brazil, 2019

During navigation, information is offered that contributes to the nurse’s guidance on the evaluation process. At the end of this process, the software provides nursing diagnoses based on NANDA-I^(^ 24 ^)^ and on the nursing interventions validated by the expert committee. All the interventions have an evidence-based justification. Photographs and step-by-step instructions for fixing and removing devices, produced by the lead researcher, are also available for access. These functionalities can be seen in Figure 3. 

Nurses are free to include or exclude interventions according to their individual assessment, the patient’s need, and the resources available at their institution. The care plan generated shows the full name, number, and state linked to the COREN of the nurse who performed the assessment. This care plan can be filed as a document in PDF format on the user’s device, sent by email, or printed on any printer that is connected with the user’s device. 

The patient’s data will be recorded only on the user’s device, which will be able to access them to perform a new evaluation in the following days. Access to the registered patients is obtained through a search field, by typing the name of the NB, the mother’s name, or the identification number. When performing the patient reassessment, the software will provide the postnatal age and the corrected GA. The weight and skin condition score based on the ECPRN will be shown in a graph, according to the patient’s subsequent assessments. Some care actions are modified as the NB grows and gains weight. The user can delete the registered data at any time.

**Figure 4:**
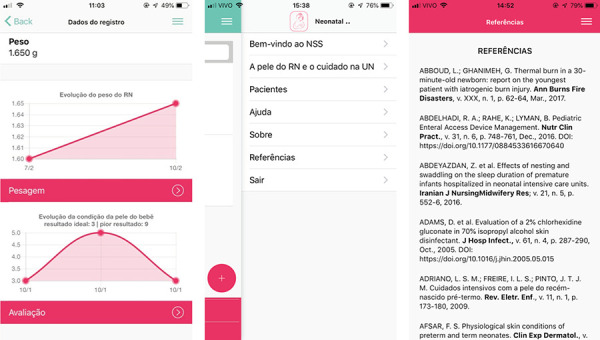
Weight graph, ECPRN score graph, and access tab to content and references - *Neonatal Skin Safe*?. Florianópolis, SC, Brazil, 2019

In a tab that can be accessed at any time during navigation, the user can return to the registered patients, get help, access information about the software, access the list of references used to prepare the content, and leave the system. In the references that present a *Digital Object Identifier*(DOI?) or an access link to *Internet*, the user has the opportunity to migrate to the platform that avails the article, being able to access it in full when it presents free access. The ECPRN weight and score graphs, the quick access tab, and the reference screen are shown in Figure 4.

For the process of evaluating the software, 43 evaluators were invited to participate in the research: 13 teaching nurses, 13 assistential nurses, 17 IT specialists. Of these, only two teacher-nurses, eight assistential nurses, and 10 IT specialists answered positively to the invitation.

The sample consisted of 20 professionals, them being 10 nurses with experience in caring for newborns and 10 specialists in the IT area. The participants were asked to return the completed instrument within seven days; however, it was necessary to extend the period by another five days, in order to ensure that the evaluators finished the process. 

In the group of nurses, all the participants were female, of these two were teacher-nurses with PhD degrees in Nursing, one was a nurse with a specialization in Stomatherapy, and seven were assistential nurses: one with a PhD, four with a Master’s degree, and two with a graduation degree. Their age varied from 25 to 38 years old, and they have worked between two and 17 years in the area. Regarding operational systems, six used Android? and four, iOS?.

In the IT group there were three women participants and seven men. Their age varied between 23 and 50 years old. Four of them are PhDs, one has a Master’s degree, and five are Bachellors in IT. Four of them have worked in the area for 25 years. The operational systems used were Android? (6) and iOS? (4).

With regard to their geographical location, the participants lived in different states of Brazil, namely: Santa Catarina, Paraná, Rio Grande do Sul, Minas Gerais, the Federal District, and Bahia.

As for the inclusion criteria, the score varied between five and 11 points in the two groups, with the teaching professionals having the highest score. 

In each group, the participants evaluated 36 key questions. These were distributed in the 23 Sub-features and six Features of Quality. Only the key question of the “capacity” sub-feature, which is part of the “performance efficiency” feature and relates to the capacity of the software’s database, was modified in the group of nurses in order to facilitate the understanding of the evaluators. 

In the assessment of the “functional adequacy” feature by the IT specialists, all the key questions obtained a concordance percentage over 70%. In the group of nurses, all the key questions of this feature presented 100% concordance.

Of the questions evaluated in the “reliability” feature, both in the IT group and in the nurses group, “fault tolerance” and “recoverability” received a concordance percentage below 70%; however, these were evaluated as “not applicable”. 

In the assessment of the “usability” feature by the nurses and by the IT specialists, only the “accessibility” sub-feature presented a percentage value below 70%. 

When the IT group assessed the “performance efficiency” feature, only the “capacity” sub-feature received 80% of “Not applicable” answers. In the group of nurses, all the questions obtained 100% concordance. 

In the IT group, the “compatibility” feature presented two questions with a concordance percentage below 70%. The highest percentage of answers was related to the “Not applicable” option. In the assessment by the nurses, all the questions obtained percentage values over 70%. 

In the assessment of the “safety” feature in the IT group, the questions related to integrity and non-rejection were assessed with less than 70%. However, they received the “Not applicable” answer. In the assessment by the nurses, all the questions obtained percentage values over 70%. 

In the evaluation of the features from the set of sub-features, excluding the “Not applicable” answers, both in the group of IT specialists and in the group of nurses, it is observed that only the “accessibility” sub-feature did not reach the appropriate percentage (> 70%) to be considered of good quality. These data are presented in Table 1.

**Table 1:** Distribution of the values according to the quality features and sub-features of *Neonatal Skin Safe* ^®^: nurses and IT specialists*. Florianópolis, SC, Brazil, 2019. (n=20)

Variable	Nurses n=10 Mean (SD^†^)	IT* n=10 Mean (SD^†^)
Functional integrity	100.0 (0.0)	100.0 (0.0)
Functional correction	100.0 (0.0)	96.7 (10.5)
Functional aptitude	100.0 (0.0)	100.0 (0.0)
**Functional adequacy**	**100.0 (0.0)**	**98.9 (3.5)**
Maturity	80.0 (42.2)	100.0 (0.0)
Fault tolerance	80.0 (42.2)	100.0 (0.0)
Recoverability	100.0 (0.0)	100.0 (0.0)
Availability	100.0 (0.0)	100.0 (0.0)
**Reliability**	**90.0 (21.1)**	**100.0 (0.0)**
Adequacy recognition	100.0 (0.0)	100.0 (0.0)
Apprehensibility	100.0 (0.0)	100.0 (0.0)
Operability	100.0 (0.0)	95.0 (15.8)
Accessibility	70.0 (48.3)	60.0 (51.6)
Error protection	90.0 (31.6)	100.0 (0.0)
User interface aesthetics	100.0 (0.0)	100.0 (0.0)
**Usability**	**93.2 (8.8)**	**85.0 (30.7)**
Time	100.0 (0.0)	100.0 (0.0)
Resources	100.0 (0.0)	100.0 (0.0)
Capacity	100.0 (0.0)	100.0 (0.0)
**Performance efficiency**	**100.0 (0.0)**	**100.0 (0.0)**
Interoperability	95.0 (15.8)	90.0 (21.1)
Coexistence	100.0 (0.0)	90.0 (31.6)
**Compatibility**	**97.5 (7.9)**	**90.0 (24.2)**
Confidentiality	80.0 (42.2)	100.0 (0.0)
Integrity	90.0 (21.1)	95.0 (15.8)
Non-rejection	100.0 (0.0)	80.0 (42.2)
Accountability	100.0 (0.0)	90.0 (31.6)
Authentication	100.0 (0.0)	90.0 (31.6)
**Safety**	**94.0 (10.7)**	**91.0 (15.2)**

* Information Technology;

† Standard Deviation

Table 1 **-** Distribution of the values according to the quality features and sub-features of *Neonatal Skin Safe*?: nurses and IT specialists?. Florianópolis, SC, Brazil, 2019. (n=20)

## Discussion

*Neonatal Skin Safe*? is a decision support system useful to equip neonatal nurses in the assessment of risks and in targeting care to prevent skin lesions in hospitalized NBs. In this sense, this technology can be seen as an important work organization strategy, in view of the fact that it streamlines the nursing process and allows for a better use of the resources available for skin care. In addition, it contributes to patient safety, preventing risks and ensuring greater quality of care. 

Computerized decision support systems offer specific recommendations and guidance to the professionals for meeting the needs and improving the patients’ health. These systems assist in solving clinical practice problems, reduce error rates, improve the accessibility of the professionals to evidence-based knowledge, and help to increase the quality and efficiency of the care provided^(^ 25 ^)^. It is also added that computerized processes are tools that optimize and simplify the actions of nurses, whether in the field of management, care or teaching, thus being related to the work process of these professionals^(^ 26 ^)^. 

The evaluation process had the participation of professionals with knowledge in neonatal nursing and in IT, allowing the software to be analyzed from different perspectives, either by looking at patient care, as well as by using technologies. Associated with this, the evaluators lived in different states of Brazil, allowing for a global view of the product and enabling the use of *Neonatal Skin Safe*? throughout the national territory. To use an instrument in different regions, it must be adapted to the local specificities of culture, language, and work of the professionals who will use it^(^ 27 ^)^.

In view of the current technological scenario, mobile devices are presented as strategies that can leverage the promotion of health education^(^ 28 ^)^. The assessment of these technologies is important, as they seek to promote improvements in safety and in the quality of care provided to patients^(^ 29 ^-^ 30 ^)^, the professionals who use them need to be sure of their quality conditions and requirements.

Being developed for mobile applications, the software is easy to access and use, allowing evaluation of the patient at the bedside. This feature guarantees greater reliability of the data collected during the evaluation process. Corroborating this issue, the use of smartphones allows for mobility breakdown, presenting the user with faster and easier access to information^(^ 31 ^)^.

The GA and weight variables, and the information on maternal HIV or Hepatitis B infection, filled-in in the patient’s registration screen, as well as the other items to be filled-in in the software during the evaluation process, are linked to various essential nursing interventions for the prevention of skin lesions in NBs. Weight and GA interfere in thermo-regulation care, in the choice of antiseptics, handling, bathing, ambience, and care with the umbilical stump, in view of the immature anatomical and physiological conditions of the skin of the newborns with GA less than 32 weeks and weight less than 1,500 grams^(^ 32 ^)^. 

In the evaluation of the “functional adequacy” feature, the difficulty of evaluation by the IT group is justified by the lack of knowledge of the evaluators, since the questions evaluated presented specificities of nursing care to the newborn, requiring mastery on this theme. However, an error was found in the inclusion and exclusion of interventions by one of the evaluators. Based on the nurses’ assessment, it is observed that the software meets the proposed objectives, promoting benefits to the care process.

The use of devices for therapy, life maintenance, and monitoring are considered the major causes of pressure injuries in neonatal patients^(^ 3 ^,^ 33 ^-^ 34 ^)^. Proper use, correct fixation, and skin protection contribute to reducing the risk of lesions^(^ 35 ^)^. In this sense, the software suggests nursing interventions, presents photographs and step-by-step instructions for properly fixing the devices, making it appealing to the user.

In the evaluation of the “usability” feature, the aesthetics of the user interface showed 100% approval by both groups. On the other hand, accessibility statistically shown to be a requirement that needs to be improved in *Neonatal Skin Safe*?. However, it is important to note that accessibility was not an aspect intended for this first version of the software. To improve this requirement, the evaluators suggested increasing the font size, the possibility of zooming the screen to assist people with visual impairments, dividing the content into several screens, and including audios and videos. It is worth highlighting that the font size can be modified in the user’s hardware itself, improving accessibility for people with reduced visual acuity. On the other hand, users with hearing or speech disabilities are not prevented from using the software, demonstrating that this result will not have a negative impact on the clinical practice.

As it does not feature a server, the data of *Neonatal Skin Safe*? are stored only locally; for this reason, the database is directly related to the user’s hardware. This justifies the IT specialists choosing “Not applicable” in the “performance efficiency” feature and in the “capacity” sub-feature.

The use of a server to store the data would allow for the patient registry to be available to all the nurses, according to their institution; this would enable the evaluation of the same NB by different professionals, ensuring continuity of the process. In addition, it would contribute to the survey of epidemiological data related to the care of the newborn’s skin. Due to financial limitations, this server was not contemplated in this research; however, it is a goal to be attained. 

In assessing the “compatibility” feature, although the software presents the possibility to access the journal online for accessing the article from the list of references, and to present the resource for sharing the care plan generated at the end of the newborn’s assessment via email or network printing, some participants claimed that they were unable to assess these requirements. This denotes that the evaluators did not use all the resources available in the software, which may have led to a lower result than expected in the evaluation of this feature.

When analyzing the data obtained in the “safety” feature, it is observed that there were disagreements in the answers of the IT specialists for some sub-features, demonstrating that, despite having been asked to carefully observe the functioning of the software, the participants did not know how to assess these questions. 

In the nurses’ evaluation, one of the evaluators had difficulties in recovering the access password; for this reason, she suggested including a register of electronic address for user and password recovery in case of forgetfulness.

In the individual assessment of the sub-features, both in the group of nurses and in the IT group, only accessibility did not reach the adequate percentage. Based on the analysis of the set of sub-features, it is observed that the results were homogeneous and all characteristics obtained a percentage above 80%, although the groups of evaluators had different experience and professional training, demonstrating that the application has excellent technical quality. It is important to note that a software application is considered of good quality when it meets the user’s needs regarding the functions, resources, and content offered^(^ 14 ^)^. In this sense, *Neonatal Skin Safe* ^®^ proved to be adequate to be widely used in the clinical practice of the neonatal team.

*Neonatal Skin Safe* ^®^ allows the nurse to conduct a global evaluation of the patient, respecting individualities and meeting each person’s needs. All the interventions that integrate the software were supported by the literature and validated by a committee of specialists in the theme, providing reliability and safety for them to be used in the care practice. The use of evidence should support and integrate the development of software targeted to health promotion, education, and care^(^ 36 ^)^. 

The learning process through mobile devices is instantaneous, occurs in an interactive way, and presents itself as a potential source of transformation of the methods of offering education and training^(^ 37 ^)^. Although developed to support decision making, the software can also be useful in the educational process of the professional. In addition to the initial screen, which contains information on anatomy, physiology, and possible risks for the development of skin lesions, all the nursing interventions have justifications based on the literature, allowing the user’s knowledge to be expanded.

In this perspective, the software can foster the critical sense of nurses, targeting actions, supporting care, reducing risks related to health care, and providing a better quality of life and safety for neonatal patients.

As limitations in carrying out this study, the following stand out: the scarcity of software that contemplate skin care of the hospitalized NB, as well as of technological evaluation studies that could serve as a model in carrying out this research. Also noteworthy is the lack of resources to contemplate a server for data storage. 

It is highlighted that all the suggestions made by the evaluators will be considered to improve the software, in order to make it even more appealing, safe, practical, and accessible for the users. It is reiterated that this action will be constant, given the accelerated process of information updates, both in terms of technological development, as well as in health care. 

As a suggestion for future research studies, it is recommended that *Neonatal Skin Safe*? be evaluated in the daily practice of nurses who care for the NBs in the NU and also by nursing students, in order to verify its effectiveness for lesion prevention, work organization, and health education.

It is expected that this study will encourage research studies on the development and evaluation of care technologies aimed at nursing and health care, collaborating with the technical scientific knowledge and with the instrumentalization of the professionals to develop their daily practice with quality and safety.

The study can be replicated in other scenarios whose theme is the evaluation of the quality of software in health care.

## Conclusion

*Neonatal Skin Safe*? is a technological innovation in health which allows the nurse to assess the risks, identify the diagnoses, and plan care actions for the prevention of skin lesions in the hospitalized NB, based on updated content, supported by the literature, and evaluated by subject matter experts. It contributes to the nurse’s work process, expands knowledge, and allows for the professional’s clinical reasoning.

This study showed that all the quality features of *Neonatal Skin Safe*? were considered as excellent, denoting that the software has the necessary technical quality to meet the needs of the nurses in addressing care actions for the prevention of skin lesions in NBs.
